# Supplementary material for: The impact of restricted provision of publicly funded elective hip and knee joints replacement during the COVID-19 pandemic in England
Source: PLoS One. 2023 Nov 29;18(11):e0294304. doi: 10.1371/journal.pone.0294304 (PMC10686417; doi:10.1371/journal.pone.0294304)
Supplement: S1 Table — (DOCX) [file pone.0294304.s001.docx]

| **Category** | **Code** | **Description** |
| --- | --- | --- |
| *Primary Total Hip Replacement* | W37.1 | Primary total prosthetic replacement of hip joint using cement |
|  | W37.8 | Other specified total prosthetic replacement of hip joint using cement |
|  | W37.9 | Unspecified total prosthetic replacement of hip joint using cement |
|  | W38.1 | Primary total prosthetic replacement of hip joint not using cement |
|  | W38.8 | Other specified total prosthetic replacement of hip joint not using cement |
|  | W38.9 | Unspecified total prosthetic replacement of hip joint not using cement |
|  | W39.1 | Primary total prosthetic replacement of hip joint NEC |
|  | W39.8 | Other specified other total prosthetic replacement of hip joint |
|  | W39.9 | Unspecified other total prosthetic replacement of hip joint |
|  | W43.1 | Primary total prosthetic replacement of other joint using cement NEC |
|  | W43.8 | Other specified total prosthetic replacement of other joint using cement NEC |
|  | W43.9 | Unspecified total prosthetic replacement of other joint using cement NEC |
|  | W44.1 | Primary total prosthetic replacement of other joint not using cement NEC |
|  | W44.8 | Other specified total prosthetic replacement of other joint not using cement NEC |
|  | W44.9 | Unspecified total prosthetic replacement of other joint not using cement NEC |
|  | W45.1 | Other primary total prosthetic replacement of other joint NEC |
|  | W45.8 | Other specified total prosthetic replacement of other joint NEC |
|  | W45.9 | Unspecified total prosthetic replacement of other joint NEC |
|  | W52.1 | Primary prosthetic replacement of articulation of bone using cement NEC |
|  | W52.8 | Other specified prosthetic replacement of articulation of bone using cement NEC |
|  | W52.9 | Unspecified prosthetic replacement of articulation of bone using cement NEC |
|  | W53.1 | Primary prosthetic replacement of articulation of bone not using cement NEC |
|  | W53.8 | Other specified prosthetic replacement of articulation of bone not using cement NEC |
|  | W53.9 | Unspecified prosthetic replacement of articulation of bone not using cement NEC |
|  | W54.1 | Primary prosthetic replacement of articulation of bone NEC |
|  | W54.8 | Other specified prosthetic replacement of articulation of bone NEC |
|  | W54.9 | Unspecified prosthetic replacement of articulation of bone NEC |
|  | W93.1 | Primary hybrid prosthetic replacement of hip joint using cemented acetabular component |
|  | W93.8 | Other specified hybrid prosthetic replacement of hip joint using cemented acetabular component |
|  | W93.9 | Unspecified hybrid prosthetic replacement of hip joint using cemented acetabular component |
|  | W94.1 | Primary hybrid prosthetic replacement of hip joint using cemented femoral component |
|  | W94.8 | Other specified hybrid prosthetic replacement of hip joint using cemented femoral component |
|  | W94.9 | Unspecified hybrid prosthetic replacement of hip joint using cemented femoral component |
|  | W95.1 | Primary hybrid prosthetic replacement of hip joint using cement NEC |
|  | W95.8 | Other specified hybrid prosthetic replacement of hip joint using cement |
|  | W95.9 | Unspecified hybrid prosthetic replacement of hip joint using cement |
| *Primary Total Knee Replacement* | W40.1 | Primary total prosthetic replacement of knee joint using cement |
|  | W40.8 | Other specified total prosthetic replacement of knee joint using cement |
|  | W40.9 | Unspecified total prosthetic replacement of knee joint using cement |
|  | W41.1 | Primary total prosthetic replacement of knee joint not using cement |
|  | W41.8 | Other specified total prosthetic replacement of knee joint not using cement |
|  | W41.9 | Unspecified total prosthetic replacement of knee joint not using cement |
|  | W42.1 | Primary total prosthetic replacement of knee joint NEC |
|  | W42.8 | Other specified other total prosthetic replacement of knee joint |
|  | W42.9 | Unspecified other total prosthetic replacement of knee joint |
|  | O18.1 | Primary hybrid prosthetic replacement of knee joint using cement |
|  | O18.8 | Other specified hybrid prosthetic replacement of knee joint using cement |
|  | O18.9 | Unspecified hybrid prosthetic replacement of knee joint using cement |
| *Resurfacing / Reconstruction* | W58.1 | Primary resurfacing arthroplasty of joint |
|  | W58.8 | Other specified reconstruction of joint |
|  | W58.9 | Unspecified other reconstruction of joint |
| *Primary unicondylar / unicompartmental knee operations* | W52.1 | Primary prosthetic replacement of articulation of bone using cement NEC |
|  | W52.8 | Other specified prosthetic replacement of articulation of other bone using cement |
|  | W52.9 | Unspecified prosthetic replacement of articulation of other bone using cement |
|  | W53.1 | Primary prosthetic replacement of articulation of bone not using cement NEC |
|  | W53.9 | Unspecified prosthetic replacement of articulation of other bone not using cement |
|  | W54.0 | Conversion from previous prosthetic replacement of articulation of bone NEC |
|  | W54.1 | Primary prosthetic replacement of articulation of bone NEC |
|  | W54.8 | Other specified other prosthetic replacement of articulation of other bone |
|  | W54.9 | Unspecified other prosthetic replacement of articulation of other bone |
