# Supplementary material for: The impact of restricted provision of publicly funded elective hip and knee joints replacement during the COVID-19 pandemic in England
Source: PLoS One. 2023 Nov 29;18(11):e0294304. doi: 10.1371/journal.pone.0294304 (PMC10686417; doi:10.1371/journal.pone.0294304)
Supplement: S3 Table — (DOCX) [file pone.0294304.s003.docx]

| **Characteristic** | **Pre COVID-19**  N = 6,044^1^ | **COVID-19 preparation**  N = 230^1^ | **COVID-19 restrictions**  N = 690^1^ |
| --- | --- | --- | --- |
| **Sex** |  |  |  |
| Female | 3,596 (59%) | 120 (52%) | 417 (60%) |
| Male | 2,448 (41%) | 110 (48%) | 273 (40%) |
| **Age On Admission^1^** | 69 (60, 77) | 66 (59, 74) | 69 (58, 77) |
| **Number of CCI conditions** |  |  |  |
| 0 | 3,319 (55%) | 115 (50%) | 325 (47%) |
| 1 | 1,924 (32%) | 78 (34%) | 247 (36%) |
| 2 | 620 (10%) | 28 (12%) | 83 (12%) |
| 3 or more | 181 (3.0%) | 9 (3.9%) | 35 (5.1%) |
| **IMD** |  |  |  |
| Least deprived | 1,620 (27%) | 70 (31%) | 205 (30%) |
| Less | 1,436 (24%) | 56 (24%) | 150 (22%) |
| Middle | 1,034 (17%) | 32 (14%) | 123 (18%) |
| More | 1,022 (17%) | 41 (18%) | 102 (15%) |
| Most deprived | 839 (14%) | 30 (13%) | 94 (14%) |
| Unknown | 93 | 1 | 16 |
| **LOS (days) ^1^** | 4.0 (3.0, 6.0) | 3.0 (2.0, 5.0) | 4.0 (3.0, 6.0) |
| **Site of surgery** |  |  |  |
| Site: hip | 3,036 (50%) | 100 (43%) | 412 (60%) |
| Site: knee | 3,008 (50%) | 130 (57%) | 278 (40%) |
| **Operation type** |  |  |  |
| Confirmed primary | 5,297 (88%) | 206 (90%) | 587 (85%) |
| Confirmed revision | 747 (12%) | 24 (10%) | 103 (15%) |
| ^1^n (%); Median (IQR) | | | |

IMD: Indices of Multiple Deprivation, CCI: Charlson Comorbidity Index, LOS: Length of stay

1 – median (25%, 75%)
