# Supplementary material for: The impact of restricted provision of publicly funded elective hip and knee joints replacement during the COVID-19 pandemic in England
Source: PLoS One. 2023 Nov 29;18(11):e0294304. doi: 10.1371/journal.pone.0294304 (PMC10686417; doi:10.1371/journal.pone.0294304)
Supplement: S4 Table — (DOCX) [file pone.0294304.s004.docx]

|  | Primary | | Revision | |
| --- | --- | --- | --- | --- |
| CCI condition | Site: hip | Site: knee | Site: hip | Site: knee |
| Myocardial infarction | 6.2% | 5.2% | 6.0% | 4.6% |
| Congestive heart failure | 3.0% | 2.2% | 1.9% | 2.6% |
| Peripheral vascular disease | 2.8% | 2.9% | 2.9% | 2.0% |
| Cerebrovascular disease | 1.2% | 1.0% | 1.0% | 0.5% |
| Dementia | 1.0% | 0.5% | 1.5% | 0.5% |
| Chronic pulmonary disease | 17.8% | 18.1% | 16.4% | 16.6% |
| Rheumatoid disease | 5.3% | 6.5% | 6.4% | 9.7% |
| Peptic ulcer disease | 0.2% | 0.3% | 0.6% | 0.0% |
| Mild liver disease | 1.5% | 1.6% | 1.2% | 2.0% |
| Diabetes without chronic complications | 11.5% | 14.0% | 11.0% | 17.9% |
| Diabetes with chronic complications | 0.6% | 0.9% | 1.2% | 0.3% |
| Hemiplegia or paraplegia | 0.3% | 0.3% | 0.2% | 0.3% |
| Renal disease | 10.0% | 8.1% | 11.0% | 8.7% |
| Cancer (any malignancy) | 1.5% | 0.9% | 1.2% | 1.0% |
| Moderate or severe liver disease | 0.1% | 0.0% | 0.2% | 0.0% |
| Metastatic cancer | 1.2% | 0.5% | 0.2% | 0.3% |
| AIDS/HIV | 0.0% | 0.0% | 0.0% | 0.0% |
