# Supplementary material for: The impact of restricted provision of publicly funded elective hip and knee joints replacement during the COVID-19 pandemic in England
Source: PLoS One. 2023 Nov 29;18(11):e0294304. doi: 10.1371/journal.pone.0294304 (PMC10686417; doi:10.1371/journal.pone.0294304)
Supplement: S5 Table — Pre COVID-19 phase restricted to operations performed on or after 01/01/2018. (DOCX) [file pone.0294304.s005.docx]

| **Characteristic** | **Confirmed primary** | | | | | **Confirmed revision** | | | | |
| --- | --- | --- | --- | --- | --- | --- | --- | --- | --- | --- |
|  | **Pre COVID-19**  N = 2,388^1^ | **COVID-19** | | | **p-value^2^** | **Pre COVID-19**  N = 361^1^ | **COVID-19** | | | **p-value^2^** |
|  |  | **Preparation**  N = 206^1^ | **Restrictions**  N = 587^1^ | **Overall**  N = 793^1^ |  |  | **Preparation**  N = 24^1^ | **Restrictions**  N = 103^1^ | **Overall**  N = 127^1^ |  |
| **Sex** |  |  |  |  | 0.456 |  |  |  |  | >0.9 |
| Female | 1,445 (61%) | 107 (52%) | 361 (61%) | 468 (59%) |  | 197 (55%) | 13 (54%) | 56 (54%) | 69 (54%) |  |
| Male | 943 (39%) | 99 (48%) | 226 (39%) | 325 (41%) |  | 164 (45%) | 11 (46%) | 47 (46%) | 58 (46%) |  |
| **Age on Admission** | 69 (59, 76) | 66 (59, 74) | 69 (57, 76) | 68 (58, 76) | 0.143 | 71 (63, 78) | 66 (61, 74) | 69 (60, 77) | 68 (60, 76) | 0.025 |
| **Number of CCI conditions** |  |  |  |  | 0.143 |  |  |  |  | 0.180 |
| 0 | 1,241 (52%) | 100 (49%) | 275 (47%) | 380 (48%) |  | 186 (52%) | 15 (62%) | 50 (49%) | 65 (51%) |  |
| 1 | 781 (33%) | 73 (35%) | 209 (36%) | 279 (35%) |  | 118 (33%) | 5 (21%) | 38 (37%) | 43 (34%) |  |
| 2 | 278 (12%) | 27 (13%) | 74 (13%) | 99 (12%) |  | 45 (12%) | 1 (4.2%) | 9 (8.7%) | 10 (7.9%) |  |
| 3 or more | 88 (3.7%) | 6 (2.9%) | 29 (4.9%) | 35 (4.4%) |  | 12 (3.3%) | 3 (12%) | 6 (5.8%) | 9 (7.1%) |  |
| **IMD** |  |  |  |  | 0.307 |  |  |  |  | 0.103 |
| Least deprived | 609 (26%) | 61 (30%) | 170 (30%) | 231 (30%) |  | 99 (28%) | 9 (38%) | 35 (35%) | 44 (35%) |  |
| Less | 557 (24%) | 52 (25%) | 131 (23%) | 183 (24%) |  | 95 (27%) | 4 (17%) | 19 (19%) | 23 (18%) |  |
| Middle | 429 (18%) | 29 (14%) | 100 (17%) | 129 (17%) |  | 62 (17%) | 3 (12%) | 23 (23%) | 26 (21%) |  |
| More | 410 (17%) | 38 (19%) | 91 (16%) | 129 (17%) |  | 60 (17%) | 3 (12%) | 11 (11%) | 14 (11%) |  |
| Most deprived | 347 (15%) | 25 (12%) | 81 (14%) | 106 (14%) |  | 39 (11%) | 5 (21%) | 13 (13%) | 18 (14%) |  |
| Unknown | 36 | 1 | 14 | 15 |  | 6 | 0 | 2 | 2 |  |
| **Surgery site** |  |  |  |  | 0.008 |  |  |  |  | 0.701 |
| Site: hip | 1,205 (50%) | 90 (44%) | 353 (60%) | 443 (56%) |  | 189 (52%) | 10 (42%) | 59 (57%) | 69 (54%) |  |
| Site: knee | 1,183 (50%) | 116 (56%) | 234 (40%) | 350 (44%) |  | 172 (48%) | 14 (58%) | 44 (43%) | 58 (46%) |  |
| **LOS (days)^3^** | 4 (3, 6) | 3 (2, 5) | 3 (3, 5) | 3 (3, 5) | <0.001 | 6 (3, 12) | 5 (2, 7) | 5 (3, 9) | 5 (3, 9) | 0.011 |
|  | - |  |  | 0.90  (0.86, 0.93) | <0.001 | - |  |  | 0.81  (0.75, 0.88) | <0.001 |
| ^1^n (%); Median (IQR)  ^2^P-values from Pearson's Chi-squared test, Wilcoxon rank sum test and Fishers exact test comparing patients treated during the ‘Pre COVID-19’ phase with those treated during the combined COVID-19 preparation and restriction phases  ^3^Incidence risk ratios, 95% confidence intervals and P-values from Poisson regression models adjusted for age, gender, IMD and number of comorbidities | | | | | | | | | | |
