# Supplementary material for: The impact of restricted provision of publicly funded elective hip and knee joints replacement during the COVID-19 pandemic in England
Source: PLoS One. 2023 Nov 29;18(11):e0294304. doi: 10.1371/journal.pone.0294304 (PMC10686417; doi:10.1371/journal.pone.0294304)
Supplement: Attachment — Submitted filename: PONE-D-22-21190_reviewer--OMR.pdf [file pone.0294304.s008.pdf]

PLOS ONE

The impact of restricted provision of elective operations during the COVID-19 pandemic on patient selection for elective NHS hip and knee replacements

--Manuscript Draft--

**Manuscript Number:**	PONE-D-22-21190
**Article Type:**	Research Article
**Full Title:**	The impact of restricted provision of elective operations during the COVID-19 pandemic on patient selection for elective NHS hip and knee replacements
**Short Title:**	Provision of elective NHS hip and knee replacements during the COVID-19 pandemic
**Corresponding Author:**	Chris M Penfold<br>University of Bristol<br>Bristol, UNITED KINGDOM
**Keywords:**	Total hip replacement, knee replacement, COVID-19
**Abstract:**	Aims<br>Elective hip and knee replacement operations were suspended in April 2020 due to the COVID-19 pandemic. The impact of this suspension and continued disruption to the delivery of joint replacement surgery is still emerging. We describe the impact of the pandemic on the provision of elective hip (HR) and knee replacements (KR) at one teaching hospital in England and on which patients had surgery.<br>Methods<br>We included all elective primary and revision HRs and KRs performed at one teaching hospital in England between 2016/01 to 2021/06. Using data from 2016-2019, we estimated the expected number of operations and beds occupied per month in 2020-2021 using time series linear models (adjusting for season and trend). We compared the predictions with the real data for 2020-2021 to assess the impact of the pandemic on the provision of surgery. We compared the length of stay and characteristics (age, gender, number of comorbidities, index of multiple deprivation) of patients who had surgery before with those who had surgery during the pandemic.<br>Results<br>We included 6,964 operations. Primary HR volume was 59% of predicted, and 47% for primary KRs. Revision HR volume was 77% of predicted, and 42% for revision KR. Median length of stay was one day shorter for primary (4 vs 3 days) and revision (6 vs 5 days) operations during the pandemic compared with before. Patients operated on during the pandemic were younger and had slightly more comorbidities than those operated on before.<br>Conclusions<br>Patients who had an elective primary or revision HR or KR during the COVID-19 pandemic were younger, had more comorbidities, but stayed in hospital for less time than those treated before the pandemic. Approximately half the number of operations were performed during the pandemic than would have been expected and the effect was greatest for revision KRs.
**Order of Authors:**	Chris M Penfold<br>Ashley Blom<br>Theresa Redaniel<br>Tim Jones<br>Emily Eyles<br>Tim Keen<br>Andrew Elliott<br>Andrew Judge
**Additional Information:**	
**Question**	**Response**

Powered by Editorial Manager® and ProduXion Manager® from Aries Systems Corporation

**Financial Disclosure**<br><br>Enter a financial disclosure statement that describes the sources of funding for the work included in this submission. Review the submission guidelines for detailed requirements. View published research articles from *PLOS ONE* for specific examples.<br><br>This statement is required for submission and **will appear in the published article** if the submission is accepted. Please make sure it is accurate.<br><br>**Unfunded studies**<br>Enter: *The author(s) received no specific funding for this work.*<br><br>**Funded studies**<br>Enter a statement with the following details:<br>• Initials of the authors who received each award<br>• Grant numbers awarded to each author<br>• The full name of each funder<br>• URL of each funder website<br>• Did the sponsors or funders play any role in the study design, data collection and analysis, decision to publish, or preparation of the manuscript?<br>• **NO** - Include this sentence at the end of your statement: *The funders had no role in study design, data collection and analysis, decision to publish, or preparation of the manuscript.*<br>• **YES** - Specify the role(s) played.<br><br>* typeset	This study was funded by the HDRUK Better Care Partnership (#6.12). This research was supported by the NIHR Biomedical Research Centre at University Hospitals Bristol and Weston NHS Foundation Trust and the University of Bristol (CP, AJ, AB) and by the National Institute for Health Research (NIHR) Applied Research Collaboration West (NIHR ARC West, MTR, TJ, EE). The views expressed in this article are those of the author(s) and not necessarily those of the NIHR or the Department of Health and Social Care.
**Competing Interests**<br><br>Use the instructions below to enter a competing interest statement for this submission. On behalf of all authors, disclose any competing interests that could be perceived to bias this work—acknowledging all financial support and any other relevant financial or non-financial competing interests.<br><br>This statement is required for submission	I have read the journal's policy and the authors of this manuscript have the following competing interests: AB (Stryker) has received research and other financial support from companies or suppliers outside the submitted work. AJ declares advisory board positions with receipt of fees (Anthera Pharmaceuticals, INC.) and paid consultancy work (Freshfields Bruckhaus Deringer) for companies outside the submitted work. All other authors declare no competing interests. This does not alter our adherence to PLOS ONE policies on sharing data and materials.

and **will appear in the published article** if the submission is accepted. Please make sure it is accurate and that any funding sources listed in your Funding Information later in the submission form are also declared in your Financial Disclosure statement.<br><br>View published research articles from *PLOS ONE* for specific examples.<br><br>**NO authors have competing interests**<br><br>Enter: *The authors have declared that no competing interests exist.*<br><br>**Authors with competing interests**<br><br>Enter competing interest details beginning with this statement:<br><br>*I have read the journal's policy and the authors of this manuscript have the following competing interests: [insert competing interests here]*<br><br>* typeset	
**Ethics Statement**<br><br>Enter an ethics statement for this submission. This statement is required if the study involved:<br><br>• Human participants<br>• Human specimens or tissue<br>• Vertebrate animals or cephalopods<br>• Vertebrate embryos or tissues<br>• Field research<br><br>Write "N/A" if the submission does not require an ethics statement.<br><br>General guidance is provided below. Consult the submission guidelines for detailed instructions. **Make sure that all information entered here is included in the Methods section of the manuscript.**	University of Bristol Faculty Research Ethics Approval was obtained. (ID #109,024)

Powered by Editorial Manager® and ProduXion Manager® from Aries Systems Corporation

**Format for specific study types**<br><br>**Human Subject Research (involving human participants and/or tissue)**<br>• Give the name of the institutional review board or ethics committee that approved the study<br>• Include the approval number and/or a statement indicating approval of this research<br>• Indicate the form of consent obtained (written/oral) or the reason that consent was not obtained (e.g. the data were analyzed anonymously)<br><br>**Animal Research (involving vertebrate animals, embryos or tissues)**<br>• Provide the name of the Institutional Animal Care and Use Committee (IACUC) or other relevant ethics board that reviewed the study protocol, and indicate whether they approved this research or granted a formal waiver of ethical approval<br>• Include an approval number if one was obtained<br>• If the study involved *non-human primates*, add *additional details* about animal welfare and steps taken to ameliorate suffering<br>• If anesthesia, euthanasia, or any kind of animal sacrifice is part of the study, include briefly which substances and/or methods were applied<br><br>**Field Research**<br><br>Include the following details if this study involves the collection of plant, animal, or other materials from a natural setting:<br>• Field permit number<br>• Name of the institution or relevant body that granted permission	
**Data Availability**<br><br>Authors are required to make all data underlying the findings described fully available, without restriction, and from the time of publication. PLOS allows rare exceptions to address legal and ethical concerns. See the PLOS Data Policy and FAQ for detailed information.	No - some restrictions will apply

Powered by Editorial Manager® and ProduXion Manager® from Aries Systems Corporation

A Data Availability Statement describing where the data can be found is required at submission. Your answers to this question constitute the Data Availability Statement and **will be published in the article**, if accepted.<br><br>**Important:** Stating 'data available on request from the author' is not sufficient. If your data are only available upon request, select 'No' for the first question and explain your exceptional situation in the text box.<br><br>Do the authors confirm that all data underlying the findings described in their manuscript are fully available without restriction?	
**Describe where the data may be found in full sentences. If you are copying our sample text, replace any instances of XXX with the appropriate details.**<br><br>• If the data are **held or will be held in a public repository**, include URLs, accession numbers or DOIs. If this information will only be available after acceptance, indicate this by ticking the box below. For example: *All XXX files are available from the XXX database (accession number(s) XXX, XXX.).*<br>• If the data are all contained **within the manuscript and/or Supporting Information files**, enter the following: *All relevant data are within the manuscript and its Supporting Information files.*<br>• If neither of these applies but you are able to provide **details of access elsewhere**, with or without limitations, please do so. For example:<br><br>*Data cannot be shared publicly because of [XXX]. Data are available from the XXX Institutional Data Access / Ethics Committee (contact via XXX) for researchers who meet the criteria for access to confidential data.*<br><br>*The data underlying the results presented in the study are available from (include the name of the third party*	The data used in the study is collected by the NBT as part of their care and support. Sharing of anonymised data with the University of Bristol was underpinned by a data sharing agreement and solely covers the purposes of this study. Data requests can be made through the NBT.

Powered by Editorial Manager® and ProduXion Manager® from Aries Systems Corporation

and contact information or URL).<br>• This text is appropriate if the data are owned by a third party and authors do not have permission to share the data.<br><br>* typeset	
Additional data availability information:	

Manuscript

Click here to access/download;Manuscript;Paper_hips and knees Covid_NBT v0.6.docx

The impact of restricted provision of elective operations during the COVID-19 pandemic on patient selection for elective NHS hip and knee replacements

Chris M Penfold[1,2,3*]

Ashley W Blom[1,2]

Maria Theresa Redaniel[3,4]

Tim Jones[3,4]

Emily Eyles[3,4]

Tim Keen[5]

Andrew Elliott[5]

Andrew Judge[1,2]

Affiliations:

1. Musculoskeletal Research Unit, Translational Health Sciences, Bristol Medical School, 1st Floor Learning & Research Building, Southmead Hospital, Bristol, BS10 5NB, UK
2. National Institute for Health Research Bristol Biomedical Research Centre, University Hospitals Bristol and Weston NHS Foundation Trust and University of Bristol, Bristol, UK
3. The National Institute for Health and Care Research Applied Research Collaboration West (NIHR ARC West) at University Hospitals Bristol and Weston NHS Foundation Trust, UK
4. Population Health Sciences, Bristol Medical School, University of Bristol, UK
5. North Bristol NHS Trust, Southmead Hospital, Westbury-on-Trym, Bristol, BS10 5NB, UK

* - denotes corresponding author

Corresponding author contact details:

Email: chris.penfold@bristol.ac.uk

Address: Musculoskeletal Research Unit, University of Bristol, School of Clinical Sciences, Learning and Research Building, Southmead Hospital, Bristol, BS10 5NB

Telephone: 0117 41 47872

Keywords
Total hip replacement, knee replacement, COVID-19

Acknowledgements
The authors are grateful to the members of the public who contributed through the patient and public involvement process and for Mike Bell and Carmel McGrath for organising the workshop.

Ethical approval: University of Bristol Faculty Research Ethics Approval was obtained. (ID #109,024)

Funding statement: This study was funded by the HDRUK Better Care Partnership (#6.12). This research was supported by the NIHR Biomedical Research Centre at University Hospitals Bristol and Weston NHS Foundation Trust and the University of Bristol and by the National Institute for Health Research (NIHR) Applied Research Collaboration West (NIHR ARC West). The views expressed in this article are those of the author(s) and not necessarily those of the NIHR or the Department of Health and Social Care.

Data sharing statement: The data used in the study is collected by the NBT as part of their care and support. Sharing of anonymised data with the University of Bristol was underpinned by a data sharing agreement and solely covers the purposes of this study. Data requests can be made through the NBT.

Author statement: This publication is the work of the authors, who serve as guarantors for the contents of this paper. CP contributed to study design, data cleaning, data analysis, interpretation of results and writing the manuscript. MTR contributed to study conceptualisation, supervision, interpretation of results and reviewing the manuscript. TK contributed to data curation, supervision, interpretation of results and reviewing the manuscript. AE contributed to data curation, interpretation of results and reviewing the manuscript. TJ and EE contributed to interpretation of results and reviewing the manuscript. AB contributed to study conceptualisation, supervision, interpretation of results and reviewing the manuscript. AJ contributed to study conceptualisation

and design, supervision, and writing the manuscript. CP had full access to the data in the study and takes responsibility for the integrity of the data and the accuracy of the data analysis.

Abstract

Aims

Elective hip and knee replacement operations were suspended in April 2020 due to the COVID-19 pandemic. The impact of this suspension and continued disruption to the delivery of joint replacement surgery is still emerging. We describe the impact of the pandemic on the provision of elective hip (HR) and knee replacements (KR) at one teaching hospital in England and on which patients had surgery.

Methods

We included all elective primary and revision HRs and KRs performed at one teaching hospital in England between 2016/01 to 2021/06. Using data from 2016-2019, we estimated the expected number of operations and beds occupied per month in 2020-2021 using time series linear models (adjusting for season and trend). We compared the predictions with the real data for 2020-2021 to assess the impact of the pandemic on the provision of surgery. We compared the length of stay and characteristics (age, gender, number of comorbidities, index of multiple deprivation) of patients who had surgery before with those who had surgery during the pandemic.

Results

We included 6,964 operations. Primary HR volume was 59% of predicted, and 47% for primary KRs. Revision HR volume was 77% of predicted, and 42% for revision KR. Median length of stay was one day shorter for primary (4 vs 3 days) and revision (6 vs 5 days) operations during the pandemic compared with before. Patients operated on during the pandemic were younger and had slightly more comorbidities than those operated on before.

Conclusions

Patients who had an elective primary or revision HR or KR during the COVID-19 pandemic were younger, had more comorbidities, but stayed in hospital for less time than those treated before the pandemic. Approximately half the number of operations were performed during the pandemic than would have been expected and the effect was greatest for revision KRs.

Introduction

The population health burden of osteoarthritis (OA) is significant and growing world-wide (1). Elective hip and knee replacements are two of the most commonly performed operations in the UK to relieve the pain and functional limitations of OA (2–4). They are effective, with ~90% of patients achieving clinically meaningful improvements in pain and function (5), have very low rates of postoperative mortality (6,7) and typical 10-year revision rates of <5% (2). The lifetime risk of hip replacement is 11.6% for women and 7.1% for men, and for knee replacement 10.8% for women and 8.1% for men (8). The mean length of hospital stay after primary hip and knee replacements is approximately three days (9).

Seasonal spikes in hospital admissions have been a priority challenge for the NHS for a number of years (10). During peak demand in 2016/17, one third of trust A&Es in England declared that hospital performance/patient safety may be compromised (11). Between 2011/12 and 2018 >£3billion was provided at short notice to support NHS services over winter (12). Management plans have been extended to include increasing bed occupancy above operating limits (85%) and cancelling elective operations (13).

In the last four years NHS England have advised wide-spread cancellations of elective operations on two occasions. The first was during the 2017/18 winter flu season when NHS England advised hospitals to suspend elective operations throughout January 2018 (14). This resulted in January-March 2018 recording the highest number of people waiting >18 weeks for their NHS treatment since quarterly records began (1994) (15). The second was due to the Covid-19 pandemic. All elective operations in the NHS were suspended from 15th April 2020 (16), initially for 'at least three months'. In reality, many hospitals had begun cancelling these operations earlier in order to increase their capacity to treat adult COVID-19 patients (17).

The impact of the COVID-19 pandemic on the delivery of elective operations is still emerging as the pandemic continues to disrupt care. In this paper we describe the impact of the COVID-19 pandemic on elective hip and knee replacements at one teaching hospital in England.

Using data from one NHS Trust we will:

1. Describe the impact of Covid-19 on the provision of elective hip and knee replacement
2. Identify inequalities in the provision of elective hip and knee replacement surgery during the Covid-19 pandemic

Methods

Setting

The setting for this study was one large teaching hospital. Before 2020, a large number of primary hip (n~650/year) and knee (n~530/year) replacement operations were performed at this hospital compared with the national average (hip: n~240/year; knee: n~270/year) and 25-40% of the total number performed in city (18).

Study sample

The Trust provided data on all NHS funded admissions for elective hip and knee primary replacement and revision operations performed at the Trust between 1st January 2016 and 30th June 2021. We included all patients who received a primary or revision hip or knee replacement operation defined by a combination of OPCS4 procedure codes and surgical site codes (Supplementary Table T1). We excluded patients who were non-elective admissions and for whom we could not unambiguously determine the site of surgery or type of operation.

The impact of restricted surgical capacity

Study timeline

The official suspension of elective operations due to the COVID-19 pandemic was on 15th April 2020. We designated this date until the end of our study period as "COVID-19 restrictions". This was preceded by hospital reorganisations to increase capacity for acute and critical care patients, meaning that elective activity was declining before officially being suspended. We have therefore designated the period from 1st January to 15th April 2020 as "COVID-19 preparation", in recognition of the disruption to normal activity. We designated the period 1st January 2016 to 31st December 2019 as "Pre COVID-19".

Change in patients being treated

We compared the characteristics of patients treated during the COVID-19 preparation and restriction periods with those treated during the 'pre COVID-19' period to understand which patients were prioritised for treatment during these periods of restricted activity.

Hospital activity

We described the number of eligible operations performed (volume of operations), the total number of beds occupied by these patients and the median number of days these patients stayed in hospital (length of stay) per calendar month.

Patient characteristics

We described the patients treated in each of the study periods using the following characteristics:

- Median age at the time of their operation in years
- Number of comorbidities
    - We counted the number of pre-existing conditions (comorbidities) patients had from those included in the Charlson comorbidity index (CCI). Comorbidities were recorded at the time of their operation using ICD-10 codes (see Supplementary Table T2 for a full list of ICD-10 codes).
    - Number of comorbidities was categorised into '0', '1', '2' and '≥3'
    - We calculated the proportion of patients operated on each month in each CCI category
- Gender
- Index of Multiple Deprivation (IMD) derived from the patient's home address
    - We categorised IMD into quintiles 'Least deprived' through to 'Most deprived'
    - We calculated the proportion of patients operated on each month in each IMD quintile

Statistical methods

Time-series modelling of elective arthroplasties

To estimate the reduction in surgical activity during the COVID-19 preparation and restriction periods we used time-series linear regression models, including terms for seasonality and overall trend, to forecast the expected monthly operation volume and bed occupancy during these periods using all available preceding activity (from 01/01/2016 to 31/12/2019). We then compared the expected and observed operation volume and bed occupancy during these periods to estimate the reduction in surgical activity. We forecast monthly operation volume and bed occupancy by primary and revision operations, and by hip and knee replacement operations.

Comparison of patient demographics and clinical characteristics

We compared the demographics and clinical characteristics of patients who received operations during the pre-COVID 19 period with those treated either in the COVID-19 preparation or COVID-19 restrictions periods, since numbers in some categories were very low. We compared characteristics using the Wilcoxon rank sum test for continuous variables (age and length of hospital stay) and Pearson's chi-square test and Fishers exact test for categorical variables. We also used Poisson regression models (adjusted for age, gender, IMD and number of comorbidities) to compare length of hospital stay.

Since we did not model the temporal trend in demographic and clinical characteristics, we were assuming that there was no change in these characteristics within each of the phases of the

pandemic. In particular, the pre-COVID 19 period covers four years, during which time demographic and clinical characteristics may have changed. We therefore repeated our comparison of these characteristics but restricted the pre-COVID 19 period to operations performed on or after 01/01/2018.

Analyses were done in R version 4.1 (19) using the 'tidyverse' suite of packages (20) and the 'comorbidity' (21), and 'forecast' (22) packages.

Results

Our study sample included 6,964 eligible operations performed between 1st January 2016 and 30th June 2021 at the Trust (Table 1). Eight hundred and seventy-four (13%) were confirmed revision operations. Between the start of our study period and the end of 2019 ('pre COVID-19') 6,044 elective hip and knee replacement operations (5,297 confirmed primaries and 747 confirmed revisions) were performed. The volume of hip and knee operations was almost identical (3,036 hip and 3,008 knee operations).

A comparison of observed versus forecast hospital activity

Two hundred and thirty operations were performed during the 'COVID-19 preparation' phase, of which 90% were confirmed primary and 10% confirmed revision operations. Since the official suspension of elective operations (15th April 2020) until the end of our study period 690 operations had been performed (Figure 1), of which 85% were confirmed primary and 15% confirmed revision operations. Using time series modelling of surgical activity up to the end of 2019, the predicted volume of elective primary and revision hip and knee replacement operations during COVID-19 preparation and restriction periods in the absence of restricted activity is 1,728 operations in total (Figure 2). The observed surgical volume was 53% of the predicted volume. The volume of knee replacement activity was more adversely affected by the COVID-19 restrictions than the volume of hip replacements, for both primary and revision operations. For primary joint replacements, the volume of knee replacements was 47.2% of predicted volume compared with 58.5% predicted volume for hip primaries. The volume of hip revision operations was 76.6% of predicted volume whereas knee revisions were only 41.7% of predicted volume. The forecast total bed occupancy during the COVID-19 preparation and restriction periods was 8,535 bed-days compared with observed occupancy of 4,393 bed-days (51.2%, Figure 3). For primary operations and hip revisions

the proportions of observed to forecast bed occupancy were similar (primary hips: 56.9%, primary knees: 52.0%, revision hips: 57.9%), but were lower for revision knees (31.6%).

A comparison of patients treated before and during the COVID-19 pandemic

In our comparison of patients treated between January 2016 and December 2019 (pre-COVID-19) with those treated during the COVID-19 preparation and restriction phases combined, we found a higher proportion of patients having a primary operation during the pandemic had 2 or more comorbidities than those treated before the pandemic (2 comorbidities: 12% versus 9.9%, ≥3 comorbidities: 4.4% versus 2.9%, P<0.001) (Table 2). A higher proportion of patients having a revision operation during the pandemic had three or more comorbidities than those treated before the pandemic (3 comorbidities: 2.7% Pre COVID-19 versus 7.1% COVID-19), but the trend was not consistent for fewer comorbidities (No comorbidities: 53% Pre COVID-19 versus 51% COVID-19, P=0.09). Revision operations were performed on younger patients during the pandemic than before (68 versus 72 years, P = 0.017). There were no differences in the sex or IMD distribution of patients treated during the pandemic compared with before the pandemic.

Median length of hospital stay was shorter during the pandemic than before for both primary (three versus four days, P<0.001, Table 2 & Figure 4) and revision operations (five versus six days, P=0.006). The adjusted Poisson regression models confirmed a reduced risk of longer hospital stays for patients treated during the pandemic compared with those before (incidence risk ratio [IRR]=0.84; 95% CI: 0.81, 0.87; P<0.001 for primaries; IRR=0.81;95% CI: 0.75, 0.86; P<0.001 for revisions). Comparisons using a shorter pre-COVID 19 time period (from 01/01/2018 onwards) showed similar results (Supplementary Table T3).

The trend towards patients with more comorbidities being treated during the pandemic persisted for primary but not revision operations.

Discussion

Patients who had an elective primary or revision hip or knee replacement operation between January 2020 and June 2021 at one large teaching hospital were younger than those treated before the COVID-19 pandemic, had more comorbidities, but also stayed in hospital for less time. We found no indication that the prioritisation of patients for surgery during the pandemic has introduced new inequalities in access to elective hip and knee replacement surgery with respect to area-level

deprivation. Overall, approximately half the number of operations were performed during this period than would have been expected without the restrictions caused by the pandemic.

We found that the volume of elective hip and knee primary replacements and revision operations between January 2020 and June 2021 was 53% of the expected volume. This is similar to the impact across England and Wales in 2020 compared with 2019 (56% of expected hips and 48% of expected knees) (23), and in other countries (24–26). We have further highlighted the concerning impact on revision operations as was observed in Poland (26), although we observed a greater impact on revisions of primary knee rather than hip replacements. Primary joint replacements may need to be revised for different reasons, including for aseptic loosening, pain, dislocation, infection, and periprosthetic fracture (2). Delays to revision operations longer than six months are likely to cause significant increases in pain and physical disability (27). Revision operations are more complex than primary joint replacements, need a longer in-hospital recovery period and are not suitable for outsourcing to the independent sector. Although these operations are much lower in volume, the reduced volume of revision operations may be of particular concern to Trusts if the trend we observed is replicated elsewhere.

We observed a reduction in the mean length of stay for primary and revision operations of one day during the pandemic compared with before. This finding was consistent using either four years or two years of pre-pandemic data, suggesting this does not reflect a pre-pandemic trend. A similar trend was reported for adults undergoing elective spinal surgery in the USA (28). This reduction may be due to the selection of patients who were more likely to need a shorter length of stay, reflected in the younger patients treated during the pandemic. But we also found that patients treated during the pandemic had more comorbidities, which is associated with longer hospital stays (29). We adjusted for patient factors, including number of comorbidities, which did not alter this finding. The shortened length of stay is unlikely to have been caused by pressure for beds from other hospital specialities but could be a consequence of infection control measures intended to minimise in-hospital transmission of COVID-19 infections. We do not know whether these patients had more post-operative complications or whether their recovery was adversely affected by their earlier discharge. If not, the safe introduction of shorter hospital stays could be a valuable finding to support the challenge of addressing the waiting list for elective hip and knee replacement operations.

Strengths and Limitations

We only have data from one NHS Trust and our findings may not generalise to other NHS Trusts. We do not have any information about activity in the independent sector and the extent to which primary operations have been outsourced from the NHS. We do not know how the waiting list has

been affected during the pandemic or the details of the patients waiting for their surgery. This was outside the scope and permissions for this study but will be an important consideration for local and national planning for the post-pandemic recovery of elective surgery provision. We have focussed on elective hip and knee replacements, but the effects of restrictions on elective operations will clearly be felt more widely. Further research looking at the impact of the COVID-19 pandemic on other elective operations may be valuable. Finally, we have not compared patient outcomes for those treated during the pandemic with those treated earlier. Surgery is likely to have been delayed for those who had their operation during the pandemic, and their experiences and outcomes may give an insight into what the outcomes might be for those still on the waiting list.

Conclusions

Despite the significant impact of the pandemic on the provision of primary and elective hip and knee replacement operations, we found no evidence of new inequalities in access to these operations. The shorter length of stay observed during the pandemic, if found to be safe and effective, may inform changes to the postoperative management of patients and influence plans to address the waiting list for these elective operations.

References

1. Vos T, Abajobir AA, Abate KH, Abbafati C, Abbas KM, Abd-Allah F, et al. Global, regional, and national incidence, prevalence, and years lived with disability for 328 diseases and injuries for 195 countries, 1990–2016: a systematic analysis for the Global Burden of Disease Study 2016. The Lancet. 2017 Sep 16;390(10100):1211–59.

2. National Joint Registry for England, Wales, Northern Ireland and the Isle of Man. NJR 18th Annual Report 2021 [Internet]. 2021 [cited 2021 Sep 22]. Available from: https://reports.njrcentre.org.uk/Portals/0/PDFdownloads/NJR%2018th%20Annual%20Report%202021.pdf

3. Ferguson RJ, Palmer AJ, Taylor A, Porter ML, Malchau H, Glyn-Jones S. Hip replacement. The Lancet. 2018 Nov 3;392(10158):1662–71.

4. Price AJ, Alvand A, Troelsen A, Katz JN, Hooper G, Gray A, et al. Knee replacement. Lancet. 2018 Nov 3;392(10158):1672–82.

5. Beswick AD, Wylde V, Gooberman-Hill R, Blom A, Dieppe P. What proportion of patients report long-term pain after total hip or knee replacement for osteoarthritis? A systematic review of prospective studies in unselected patients. BMJ Open. 2012;2(1):e000435.

6. Hunt LP, Ben-Shlomo Y, Clark EM, Dieppe P, Judge A, MacGregor AJ, et al. 45-day mortality after 467 779 knee replacements for osteoarthritis from the National Joint Registry for England and Wales: an observational study. The Lancet. 2014 Oct 18;384(9952):1429–36.

7. Hunt LP, Ben-Shlomo Y, Clark EM, Dieppe P, Judge A, MacGregor AJ, et al. 90-day mortality after 409 096 total hip replacements for osteoarthritis, from the National Joint Registry for England and Wales: A retrospective analysis. Lancet. 2013;382(9898):1097–104.

8. Culliford DJ, Maskell J, Kiran A, Judge A, Javaid MK, Cooper C, et al. The lifetime risk of total hip and knee arthroplasty: results from the UK general practice research database. Osteoarthritis Cartilage. 2012 Jun;20(6):519–24.

9. Garriga C, Murphy J, Leal J, Arden NK, Price AJ, Prieto-Alhambra D, et al. Assessment on patient outcomes of primary hip replacement: an interrupted time series analysis from 'The National Joint Registry of England and Wales'. BMJ Open. 2019 Nov 1;9(11):e031599.

10. BMA - Beds in the NHS [Internet]. [cited 2020 Jan 14]. Available from: https://www.bma.org.uk/collective-voice/policy-and-research/nhs-structure-and-delivery/monitoring-quality-in-the-nhs/beds-in-the-nhs

11. Hunter S. Operational Pressures Escalation Levels Framework. NHS England; 2016 Oct p. 22.

12. A short history of NHS winter funding [Internet]. The King's Fund. 2018 [cited 2019 Dec 20]. Available from: https://www.kingsfund.org.uk/blog/2018/10/short-history-nhs-winter-funding

13. Iacobucci G. Hospitals are using "escalation" beds routinely, not just in winter, BMA finds. BMJ [Internet]. 2019 Jun 21 [cited 2019 Dec 18];365. Available from: https://www-bmj-com.bris.idm.oclc.org/content/365/bmj.l4364

14. Iacobucci G. NHS cancels planned surgery and outpatient appointments in response to winter crisis. BMJ. 2018 Jan 3;k19.

15. Iacobucci G. NHS waiting times: number of patients waiting 18 weeks for treatment rises sharply. BMJ. 2018 May 11;361:k2114.

16. Iacobucci G. Covid-19: all non-urgent elective surgery is suspended for at least three months in England. BMJ. 2020 Mar 18;m1106.

17. Zahra W, Dixon JW, Mirtorabi N, Rolton DJ, Tayton ER, Hale PC, et al. Safety evaluation of a strategy to restart elective orthopaedic surgery during the de-escalation phase of the COVID-19 pandemic. Bone & Joint Open. 2020 Aug;1(8):450–6.

18. National Joint Registry. Hospital Profile - NJR Surgeon and Hospital Profile [Internet]. [cited 2021 Jan 15]. Available from: https://surgeonprofile.njrcentre.org.uk/

19. R Core Team. R: A Language and Environment for Statistical Computing [Internet]. Vienna, Austria: R Foundation for Statistical Computing; 2018. Available from: https://www.R-project.org/

20. Wickham H, Averick M, Bryan J, Chang W, McGowan LD, François R, et al. Welcome to the Tidyverse. Journal of Open Source Software. 2019 Nov 21;4(43):1686.

21. Gasparini A. comorbidity: An R package for computing comorbidity scores. Journal of Open Source Software. 2018 Mar 30;3(23):648.

22. Hyndman RJ, Khandakar Y. Automatic Time Series Forecasting: The forecast Package for R. Journal of Statistical Software. 2008 Jul 29;27:1–22.

23. Sayers A, Deere K, Lenguerrand E, Kunutsor SK, Rees JL, Judge A, et al. The COVID-19 induced joint replacement deficit in England, Wales and Northern Ireland [Internet]. The National Joint Registry 18th Annual Report 2021 [Internet]. National Joint Registry; 2021 [cited 2022 Jan 26]. Available from: https://www.ncbi.nlm.nih.gov/books/NBK576854/

24. Heckmann ND, Bouz GJ, Piple AS, Chung BC, Wang JC, Mayfield CK, et al. Elective Inpatient Total Joint Arthroplasty Case Volume in the United States in 2020: Effects of the COVID-19 Pandemic. JBJS. :10.2106/JBJS.21.00833.

25. Yapp LZ, Clarke JV, Moran M, Simpson AHRW, Scott CEH. National operating volume for primary hip and knee arthroplasty in the COVID-19 era: a study utilizing the Scottish arthroplasty project dataset. Bone & Joint Open. 2021 Mar;2(3):203–10.

26. Czubak-Wrzosek M, Czubak J, Grzelecki D, Tyrakowski M. The Effect of the COVID-19 Pandemic on Total Hip and Knee Arthroplasty Surgical Volume in 2020 in Poland. IJERPH. 2021 Aug 21;18(16):8830.

27. Davis AM, Agnidis Z, Badley E, Davey JR, Gafni A, Gollish J, et al. Waiting for hip revision surgery: the impact on patient disability. Can J Surg. 2008 Apr;51(2):92–6.

28. Wang KY, McNeely EL, Dhanjani SA, Raad M, Puvanesarajah V, Neuman BJ, et al. COVID-19 Significantly Impacted Hospital Length of Stay and Discharge Patterns for Adult Spinal Deformity Patients. Spine (Phila Pa 1976). 2021 Nov 15;46(22):1551–6.

29. Olthof M, Stevens M, Bulstra SK, Akker-Scheek I van den. The association between comorbidity and length of hospital stay and costs in total hip arthroplasty patients: a systematic review. The Journal of Arthroplasty. 2014 May 1;29(5):1009–14.

Figure captions

Figure 1: Annotated time-series of elective total hip replacements and knee replacements performed per month, 2016 to 2021

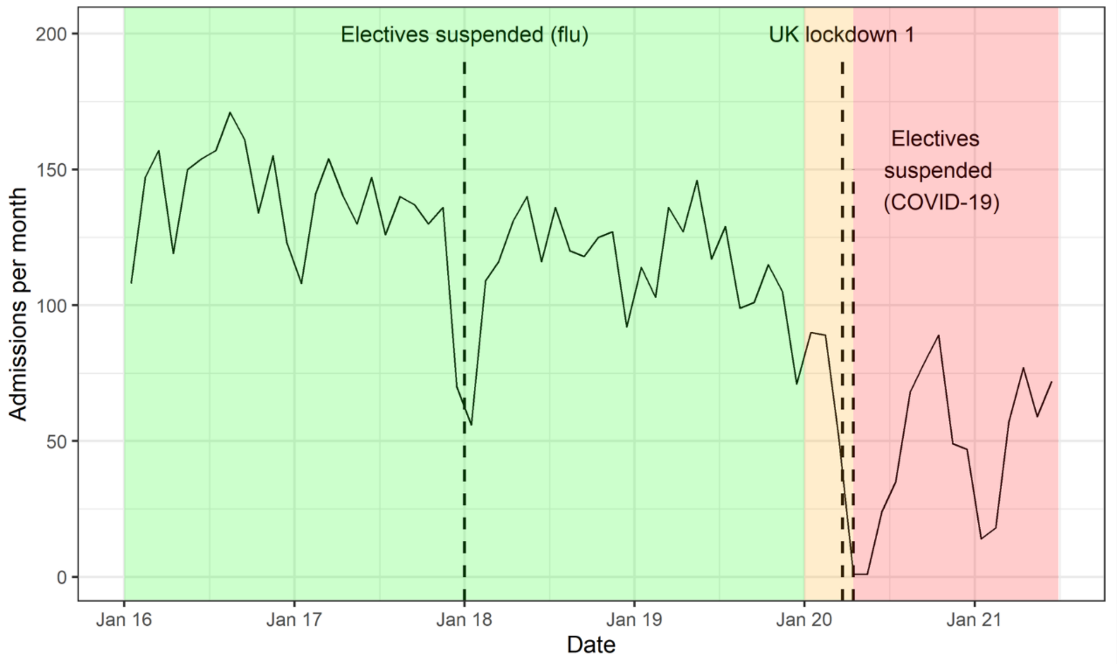

Note – background colours signify phases of the COVID-19 pandemic: green = 'Pre COVID-19', orange = 'COVID-19 preparation', red = 'COVID-19 restrictions'

Figure 2: A comparison of the observed volume of elective hip and knee replacements 2016-2019 (solid black) compared with the forecast volume (solid red) and observed volume (dashed black) between 1st January 2020 and 30th June 2021 per calendar month, by operation (primary or revision joint replacement operation) and location (hip or knee)

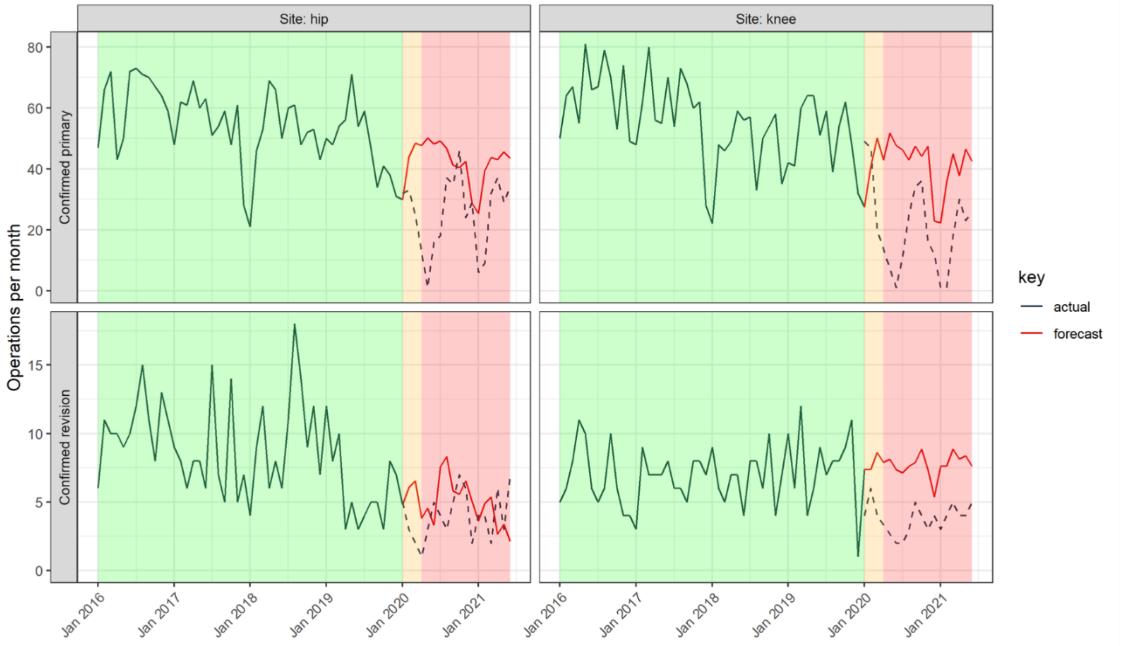

Note – background colours signify phases of the COVID-19 pandemic: green = 'Pre COVID-19', orange = 'COVID-19 preparation', red = 'COVID-19 restrictions'

Figure 3: A comparison of the observed bed occupancy by patients receiving primary and revision elective hip and knee replacements 2016-2019 (solid black) compared with the forecast (solid red) and observed bed occupancy (dashed black) between 1st January 2020 and 30th June 2021 per calendar month, by operation (primary or revision joint replacement operation) and location (hip or knee)

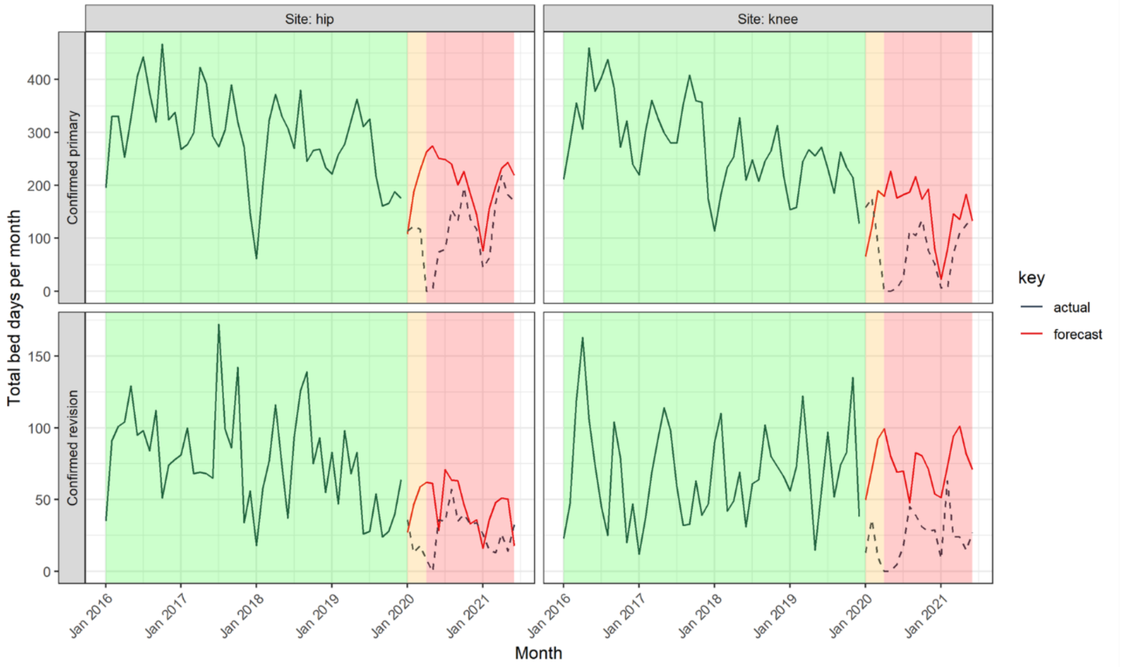

Note – background colours signify phases of the COVID-19 pandemic: green = ‘Pre COVID-19’, orange = ‘COVID-19 preparation’, red = ‘COVID-19 restrictions’

Figure 4: Median length of stay by operation type and site of surgery

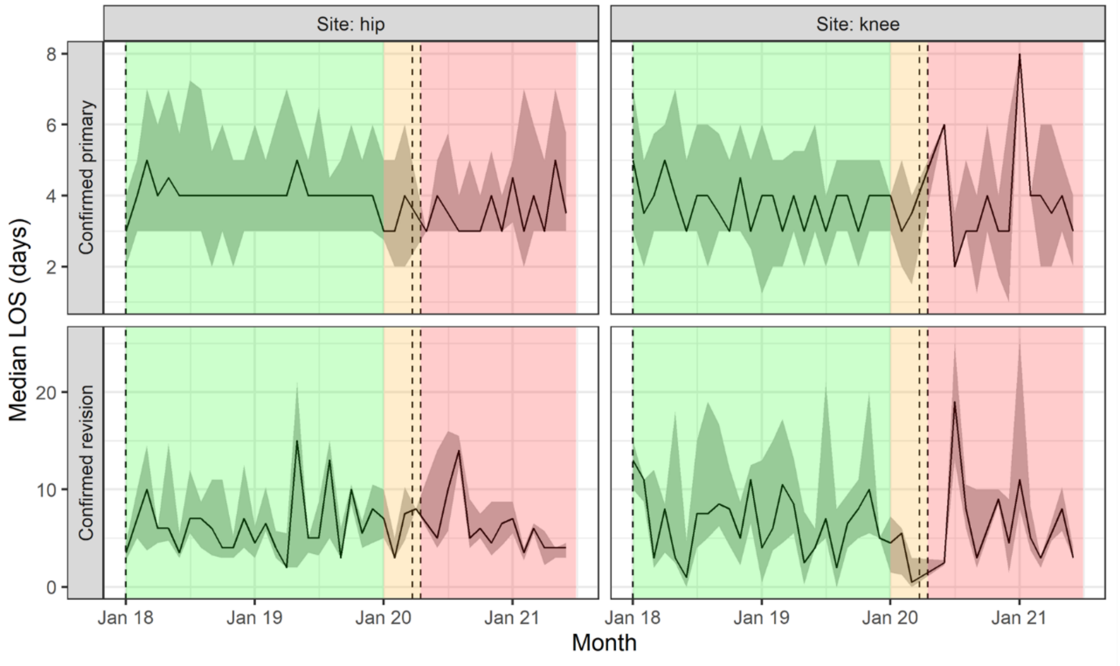

Note – background colours signify phases of the COVID-19 pandemic: green = 'Pre COVID-19', orange = 'COVID-19 preparation', red = 'COVID-19 restrictions'

Table 1:A description of the study sample by COVID-19 phase

Characteristic	Pre COVID-19<br>N = 6,044[1]	COVID-19 preparation<br>N = 230[1]	COVID-19 restrictions<br>N = 690[1]
**sex**			
Female	3,596 (59%)	120 (52%)	417 (60%)
Male	2,448 (41%)	110 (48%)	273 (40%)
**Age On Admission**	69 (60, 77)	66 (59, 74)	69 (58, 77)
**Number of comorbidities**			
0	3,380 (56%)	115 (50%)	330 (48%)
1	1,881 (31%)	79 (34%)	243 (35%)
2	607 (10%)	27 (12%)	82 (12%)
≥3	176 (2.9%)	9 (3.9%)	35 (5.1%)
**IMD**			
Least deprived	1,620 (27%)	70 (31%)	205 (30%)
Less	1,436 (24%)	56 (24%)	150 (22%)
Middle	1,034 (17%)	32 (14%)	123 (18%)
More	1,022 (17%)	41 (18%)	102 (15%)
Most deprived	839 (14%)	30 (13%)	94 (14%)
Unknown	93	1	16
**LOS (days)**	4.0 (3.0, 6.0)	3.0 (2.0, 5.0)	4.0 (3.0, 6.0)
**Site of surgery**			
Site: hip	3,036 (50%)	100 (43%)	412 (60%)
Site: knee	3,008 (50%)	130 (57%)	278 (40%)
**Operation type**			
Confirmed primary	5,297 (88%)	206 (90%)	587 (85%)
Confirmed revision	747 (12%)	24 (10%)	103 (15%)

[1]n (%); Median (IQR)

Table 2: A description of the study sample by COVID-19 phase and operation type

Characteristic	Confirmed primary: Pre COVID-19 N = 5,297[1]	Confirmed primary: COVID-19 Preparation N = 206[1]	Confirmed primary: COVID-19 Restrictions N = 587[1]	Confirmed primary: COVID-19 Overall N = 793[1]	p-value[2]	Confirmed revision: Pre COVID-19 N = 747[1]	Confirmed revision: COVID-19 Preparation N = 24[1]	Confirmed revision: COVID-19 Restrictions N = 103[1]	Confirmed revision: COVID-19 Overall N = 127[1]	p-value[2]
**Sex**					0.5					0.8
Female	3,197 (60%)	107 (52%)	361 (61%)	468 (59%)		399 (53%)	13 (54%)	56 (54%)	69 (54%)	
Male	2,100 (40%)	99 (48%)	226 (39%)	325 (41%)		348 (47%)	11 (46%)	47 (46%)	58 (46%)	
**Age on Admission**	69 (59, 76)	66 (59, 74)	69 (57, 76)	68 (58, 76)	0.082	72 (63, 78)	66 (61, 74)	69 (60, 77)	68 (60, 76)	0.017
**Number of CCI conditions**					<0.001					0.090
0	2,984 (56%)	100 (49%)	280 (48%)	380 (48%)		396 (53%)	15 (62%)	50 (49%)	65 (51%)	
1	1,630 (31%)	74 (36%)	205 (35%)	279 (35%)		251 (34%)	5 (21%)	38 (37%)	43 (34%)	
2	527 (9.9%)	26 (13%)	73 (12%)	99 (12%)		80 (11%)	1 (4.2%)	9 (8.7%)	10 (7.9%)	
>=3	156 (2.9%)	6 (2.9%)	29 (4.9%)	35 (4.4%)		20 (2.7%)	3 (12%)	6 (5.8%)	9 (7.1%)	
**IMD**					0.7					0.076
Least deprived	1,424 (27%)	61 (30%)	170 (30%)	231 (30%)		196 (27%)	9 (38%)	35 (35%)	44 (35%)	
Less	1,248 (24%)	52 (25%)	131 (23%)	183 (24%)		188 (26%)	4 (17%)	19 (19%)	23 (18%)	
Middle	889 (17%)	29 (14%)	100 (17%)	129 (17%)		145 (20%)	3 (12%)	23 (23%)	26 (21%)	

More	897 (17%)	38 (19%)	91 (16%)	129 (17%)		125 (17%)	3 (12%)	11 (11%)	14 (11%)	
Most deprived	759 (15%)	25 (12%)	81 (14%)	106 (14%)		80 (11%)	5 (21%)	13 (13%)	18 (14%)	
Unknown	80	1	14	15		13	0	2	2	
**Surgery site**					<0.001					0.8
Site: hip	2,623 (50%)	90 (44%)	353 (60%)	443 (56%)		413 (55%)	10 (42%)	59 (57%)	69 (54%)	
Site: knee	2,674 (50%)	116 (56%)	234 (40%)	350 (44%)		334 (45%)	14 (58%)	44 (43%)	58 (46%)	
**LOS (days)**	4 (3, 6)	3 (2, 5)	3 (3, 5)	3 (3, 5)	<0.001[2]	6 (4, 12)	5 (2, 7)	5 (3, 9)	5 (3, 9)	0.006[2]
	-			0.84 (0.81, 0.87)[3]	<0.001[3]	-			0.81 (0.75, 0.86)[3]	<0.001[3]

[1]n (%); Median (IQR)

[2]P-values from Pearson's Chi-squared test, Wilcoxon rank sum test and Fishers exact test comparing patients treated during the ‘Pre COVID-19’ phase with those treated during the combined COVID-19 preparation and restriction phases

[3]Incidence risk ratios, 95% confidence intervals and P-values from Poisson regression models adjusted for age, gender, IMD and number of comorbidities

Supplementary material

Click here to access/download
Supporting Information
Paper_hips and knees Covid_NBT v0.6_suppl.docx
