# Supplementary material for: The impact of restricted provision of publicly funded elective hip and knee joints replacement during the COVID-19 pandemic in England
Source: PLoS One. 2023 Nov 29;18(11):e0294304. doi: 10.1371/journal.pone.0294304 (PMC10686417; doi:10.1371/journal.pone.0294304)
Supplement: Attachment — Submitted filename: Comments.docx [file pone.0294304.s006.docx]

Dear Sir,

In general, the subject of the manuscript is very good besides the authors used the suitable statistical analysis but I have some comments

1.The authors mentioned that since numbers in some categories were very low. They compared characteristics using the Wilcoxon rank sum test for continuous variables (age and length of hospital stay). This is not an accurate paragraph. The Wilcoxon rank sum test should be used when the data are not normally distributed. Otherwise, the independent t-test is the preferred test.

2.The means of age and length of hospital need to adding the standard deviation

3.The paper included a p-value as one number after the dot and I think adding two numbers is more suitable.

4- I think it is preferred for the authors to state that they used the Chi-square test of independence to determine whether there is an association between categorical variables (i.e., whether the variables are independent or related).

5. It is better for authors to include the equations of time-series linear regression models in the paper in order not to waste their efforts
