# Supplementary material for: The impact of restricted provision of publicly funded elective hip and knee joints replacement during the COVID-19 pandemic in England
Source: PLoS One. 2023 Nov 29;18(11):e0294304. doi: 10.1371/journal.pone.0294304 (PMC10686417; doi:10.1371/journal.pone.0294304)
Supplement: Attachment — Submitted filename: PLOS ONE paper review 14.docx [file pone.0294304.s007.docx]

**Manuscript Number: PONE-D-22-21190**

Re “The impact of restricted provision of elective operations during the COVID-19 pandemic on patient selection for elective NHS hip and knee replacements”

**Thank you for inviting me to review this manuscript; I have the followings Comments:**

**Introduction:** the statement “Seasonal spikes in hospital admissions…” Explain and support it with data”

The objectives of the study should be rewritten to be more specific and relevant to the outcome of the study rather than describe.

**Methods:**

How were the data extracted?

What was the sampling technique?

The statement under setting “ (hip: n~240/year; knee: n~270/year) and 25-40% of the total number performed in city” Which city?

Explain CCI, IMD and comment on their validity and these must be supported with references.

The statement “Since we did not model the temporal trend in demographic and clinical characteristics, we were assuming that there was no change in these characteristics within each of the phases of the pandemic” It should be moved under limitations of the study. Furthermore, assumption was inappropriate to mention in this study, please revise.

**Tables:** Explain in the text the demography of the participants shown in Table 1 **.**

What was IMD stand for in Table 1?. This was mentioned in the text, and it should be also inserted under Table 1.

What were CCI and LOS stand for in Table 2?. These were mentioned in the text and it should be also inserted under Table 2. Also, In the text, give examples of comorbidities mentioned in Table 2.

Explain the main findings of figures 1, 2 and 3.

Explain the findings of p-values, for example P=0.09, P=0. 017..etc.

The statement in the last lines of results “The trend towards patients with more comorbidities being treated during the pandemic persisted for primary but not revision operations.” Was this investigated in this study or was it an assumption”

**Discussion:**

It should be revised, and authors should mention each finding rather than listing all findings once and then contrast each with relevant published studies. For example authors quoted “….elective spinal surgery….” under reference 28 which was irrelevant to knee and or hip surgery.

Under discussion, the statement “We observed a reduction in the mean length of stay for primary and revision operations of one day during the pandemic compared with before.” Was this investigated in this study as it was not mentioned under the results or Tables of this study.

**Strengths and Limitations:**

Start with the strengths of your study as nothing in this regard was mentioned.

**Conclusions:**

The statement “……. we found no evidence of new inequalities in access to these operations” Was this investigated in the study? If yes it should be highlight and explained under the results of this study.

**General comments:**

Ethics statement should be explained in detail.

This study will benefit from two lines of recommendations.
